# Is hyperoxia during veno-arterial extracorporeal life support due to cardiopulmonary failure associated with mortality in pediatric patients?

**DOI:** 10.1051/ject/2025006

**Published:** 2025-09-15

**Authors:** Asaad G. Beshish, Rebecca Shamah, Joshua Qian, Kasey Keane-Lerner, Paola Rodriguez Morales, Tawanda Zinyandu, Joel Davis, Joshua M. Rosenblum, Heather K. Viamonte

**Affiliations:** 1 Department of Pediatrics, Division of Cardiology, Emory University School of Medicine, Children’s Healthcare of Atlanta Atlanta GA 30329 USA; 2 Emory University School of Medicine Atlanta GA 30329 USA; 3 Physician Assistant, Children’s Healthcare of Atlanta Atlanta GA 30329 USA; 4 Senior Research Coordinator, Children’s Healthcare of Atlanta Atlanta GA 30329 USA; 5 Advanced Technology Coordinator, ECMO and Advanced Technologies, Children’s Healthcare of Atlanta Atlanta GA 30329 USA; 6 Department of Surgery, Division of Cardiothoracic Surgery, Emory University School of Medicine, Children’s Healthcare of Atlanta Atlanta GA 30329 USA

**Keywords:** Extracorporeal Life Support (ECLS), Hyperoxia, Veno-arterial Extracorporeal Life Support (VA-ECLS), Mortality, Functional Status Scale (FSS)

## Abstract

*Background*: Data is limited regarding the effects of supraphysiologic blood oxygen tension in patients requiring extracorporeal life support (ECLS). We sought to evaluate the association between hyperoxia and outcomes in pediatric patients requiring veno-arterial (VA) ECLS. *Methods*: Retrospective single-center study at an academic children’s hospital that included all patients 0–18 years who required VA-ECLS between 01/2014 and 12/2019. *Results*: During the study period, 229 VA-ECLS runs occurred in 229 patients. The majority of patients were neonates (73.4%), with cardiac being the most common indication (48.9%). The median time from admission to cannulation was 78.5 h (IQR 14, 356) with a median ECLS duration of 111.5 h (IQR 65.5, 184.5). The overall mortality rate was 44.5%. Using a receiver operating curve, a mean PaO_2_ of 233 mmHg in the first 48 h of ECLS was determined to have the optimal discriminatory ability for mortality (sensitivity 36% and specificity 76%). Of the VA-ECLS cohort, 68 (29.7%) had a mean PaO_2_ > 233 mmHg (hyperoxia group). The hyperoxia group tended to be older (median age 4.6 vs 1.5 months, *p* = 0.019), had a primary cardiac indication for VA-ECLS (60% vs 44%, *p* = 0.0004), and had a higher mortality rate (54% vs 40%, *p* = 0.050). In the multivariable analysis, after adjusting for covariables, the data demonstrated increased odds of mortality (aOR 2.02, 95% CI [1.03, 3.97], *p* = 0.03). The odds of development of stage II or III acute kidney injury (AKI) (aOR 2.04, 95% CI [0.82, 5.50]), but that did not reach statistical significance (*p* = 0.120). *Conclusion*: There is evidence that hyperoxia during the first 48 h of VA-ECLS may be associated with mortality and development of acute kidney injury, although this did not reach statistical significance. Multicenter and prospective evaluation of this modifiable risk factor is imperative to improve the care of this high-risk cohort.

## Introduction

Extracorporeal life support (ECLS) is commonly used to support patients with reversible cardiopulmonary failure refractory to conventional medical treatment. While the primary purpose of ECLS is bridge-to-recovery, it has also been deployed as a bridge-to-bridge, bridge-to-transplantation, or bridge-to-decision [1, 2]. Since the introduction of ECLS in the mid-1970s, its utilization to support patients with complex pathologies has steadily risen. According to the most recent ELSO registry report in 2024, over 89,000 neonatal and pediatric patients have been supported with ECLS, with an overall survival rate of about 53% [3].

During veno-arterial (VA-) ECLS, deoxygenated blood is drained from the venous side of the circulation, oxygenated, and pumped back to the arterial side. ECLS circuits utilize a highly efficient oxygenator whereby the arterial blood returning to the body contains high partial pressure of oxygen (PaO_2_) that can exceed 400–500 mmHg. When cells are exposed to supraphysiologic levels of oxygen in the bloodstream, the term hyperoxia is used. Hyperoxia has been well studied in various clinical scenarios, including after resuscitation from cardiac arrest (CA), perinatal asphyxia, myocardial infarction, traumatic brain injury, and following cardiopulmonary bypass (CPB). Several studies in both adults and children have demonstrated an association between hyperoxia exposure and increased morbidity and mortality [2, 4–14]. Although the association of hyperoxia with adverse outcomes has been shown previously, the level at which PaO_2_ becomes deleterious may differ depending on the clinical situation, including the duration of exposure, the patient’s age, underlying physiology, and disease process [4, 5, 9, 13–17].

Given the lack of a clear definition of hyperoxia, we sought to evaluate hyperoxia exposure in a high-risk patient population who required ECLS for cardiopulmonary failure. Our primary aim was to determine if hyperoxia while on ECLS was associated with mortality using a derived cut-point within our cohort. Our secondary aim was to determine if hyperoxia during ECLS was associated with greater odds of new morbidity including Functional Status Scale (FSS), and the development of complications while on ECLS, such as acute kidney injury (AKI).

## Materials and methods

This is a single-center retrospective cohort study in a high-volume ECLS center. All patients who required VA-ECLS due to cardiopulmonary failure between January 1, 2014, and December 31, 2019, at Children’s Healthcare of Atlanta (CHOA), a free-standing, university-affiliated quaternary children’s hospital. An internal ECLS database was queried, and eligible patient encounters were identified. The study was approved by the CHOA Institutional Review Board (IRB# 00001239, approval date: 10/11/2022). Informed consent was waived.

### Data and definitions

All consecutive patients who required VA-ECLS support in index hospitalization were included. Demographic features, clinical characteristics, and ECLS variables were collected. All arterial blood gases were obtained from the patient’s arterial line during the first 48 h while on ECLS. The primary outcome was defined as all-cause ECLS mortality. The secondary outcome variables included FSS, AKI (Stage II or Stage III, as defined by the KDIGO criteria) [18] and major complications. Major complications were defined as the presence of either cardiovascular, renal or mechanical complications.

### Functional Status Scale (FSS)

The FSS consists of six main domains: mental status, sensory, communication, motor function, feeding, and respiratory. Functional status for each domain was categorized from a normal score of 1 to very severe dysfunction with a score of 5, giving total FSS scores ranging from 6 to 30 as previously described [19]. Functional status scoring for this study involved retrospectively scoring baseline status (prior to admission functional status according to caregivers) and again at hospital discharge by examining the detailed history and physical exam performed by the primary medical team. FSS score determination was blinded from hyperoxia status. Newborns who had never achieved a stable baseline FSS were assigned a score of 6. This was operationalized by assigning a baseline FSS score of 6 to all admissions for infants 0–2 days old and to transfers from another facility for infants 3–6 days old, as previously reported [20–23]. New morbidity was defined as an increase in total FSS score of ≥3 points, and unfavorable functional outcomes were defined as an increase of ≥5 [24].

### Clinical management

All circuits were blood primed before the start of ECLS with packed red blood cells, 25% albumin, sodium bicarbonate, calcium gluconate, and heparin for patients <40 kg. It is common practice for ABGs to be obtained at the discretion of the clinical team, most typically 30 min after initial ECLS-cannulation, and then hourly for the first 3 h. Subsequently, they are typically obtained every 3–6 h and 30 min after an adjustment in ECLS support. Target gas exchange parameters are not dictated by protocol at our center. Goal PaO_2_ ranges have not established as normal and the variation we describe is derived from measurements occurring during clinical care. Goal PaCO_2_ was 35–45 mmHg, and goal pH was 7.35–7.45. Once patients are placed on ECLS, the ventilator is placed on “rest settings” of the following: ventilator mode pressure control, peak inspiratory pressure 20 cm H_2_O, peak end-expiratory pressure 10 cm H_2_O, respiratory rate 20/minute, inspiratory time 1 second, and FiO_2_ 30%.

### Statistical analysis

Statistical analysis was conducted using SAS version 9.0 software, with a significance level set at *p* < 0.05. The diagnostic utility of mean PaO_2_ in predicting mortality was evaluated using Youden’s index *(J* = sensitivity + specificity − 1) and receiver operating characteristic (ROC) curves. The study population was stratified into hyperoxia and non-hyperoxia groups based on the optimal cut-off value for mean PaO_2_, determined by maximizing the *J*-value. Fisher’s exact test was employed for comparing categorical variables, while Student’s t-test and the nonparametric Wilcoxon rank-sum test were used for continuous variables, as appropriate. Additionally, a scatterplot was generated to examine the relationship between mean PaO_2_, duration of ECLS run, and survival, with Spearman’s correlation coefficient reported. To assess the impact of hyperoxia on mortality and AKI, univariable and multivariable logistic regression analyses were performed, adjusting for BSA, age group, and indication for ECLS in the multivariable analysis, which were determined a priori. The results are presented as odds ratios (OR) with corresponding 95% confidence intervals (CI).

## Results

During the study period, 229 VA-ECLS runs occurred in 229 patients. The median age of this cohort was 2.5 months (IQR 0.3, 19.0) with a weight of 4.4 kg (IQR 3.2, 10.7), and an even distribution of males (49.8%) and females (50.2%). The majority were neonates (73.4%), with primary cardiac failure being the most common indication (48.9%) for ECLS. The median time from admission to cannulation was 78.5 h (IQR 14, 356) with a median run duration of 111.5 h (IQR 65.5, 184.5). The overall mortality rate was 44.5% (Table 1). There was a total of 3664 PaO_2_ samples obtained for the entire cohort. The range of samples per patient was 11–32, and the median number of samples per patient was 17 (IQR 14, 21).

**Table 1:** Patient demographics and clinical characteristics of entire VA-ECLS cohort stratified by mean PaO_2_ levels in the first 48 h into non-hyperoxia group (PaO_2_ ≤ 233 mmHg) hyperoxia group (PaO_2_ > 233 mmHg).

Variables	Total Cohort	Non-hyperoxia group	Hyperoxia group	*p*-value
	(*n* = 229)	(PaO_2_ ≤ 233 mmHg) (*n* = 161)	(PaO_2_ 233 mmHg) (*n* = 68)	
Age (months)	2.5 (0.3, 19.0)	1.5 (0.2, 17.2)	4.6 (0.4, 27.8)	0.019
Age Group				0.345
Neonatal	168 (73.4%)	121 (75.2%)	47 (69.1%)	
Pediatrics	61 (26.6%)	40 (24.8%)	21 (30.9%)	
Weight (kg)	4.4 (3.2, 10.7)	3.9 (3.1, 9.5)	5.5 (3.3, 11.9)	0.178
Height (cm)	54.0 (49.0, 80.0)	53.0 (49.0, 75.5)	61.8 (50.0, 84.3)	0.115
BSA (m^2^)	0.3 (0.2, 0.5)	0.2 (0.2, 0.4)	0.3 (0.2, 0.5)	0.155
Race				0.204
Black	104 (45.4%)	75 (46.6%)	29 (42.6%)	
White	87 (38.0%)	55 (34.2%)	32 (47.1%)	
Hispanic	26 (11.4%)	21 (13.0%)	5 (7.4%)	
Other	12 (5.2%)	10 (6.2%)	2 (2.9%)	
Sex				0.410
Female	115 (50.2%)	78 (48.4%)	37 (54.4%)	
Male	114 (49.8%)	83 (51.6%)	31 (45.6%)	
ECLS indication				0.0004
Cardiac	112 (48.9%)	71 (44.1%)	41 (60.3%)	
ECPR	65 (28.4%)	42 (26.1%)	23 (33.8%)	
Pulmonary	52 (22.7%)	48 (29.8%)	4 (5.9%)	
Time from admission to ECLS Initiation	78.5 (14.0, 356.0)	72.5 (14.0, 411.0)	100.0 (7.0, 306.0)	0.997
Initial ECLS flow (mL/kg/min)	114 (94, 116)	109 (100, 1212)	128 (97, 131)	0.018
Duration of ECLS run (hours)	111.5 (65.5, 184.5)	111.5 (65.5, 195.5)	110.5 (66.5, 161.5)	0.412
ECLS Complications:				
Cardiovascular	87 (45.8%)	51 (39.8%)	36 (58.1%)	0.018
Hemorrhagic	86 (45.3%)	58 (45.3%)	28 (45.2%)	0.984
Mechanical	75 (39.5%)	58 (45.3%)	17 (27.4%)	0.018
Renal	103 (54.2%)	68 (53.1%)	35 (56.5%)	0.666
Neurologic	46 (24.2%)	28 (21.9%)	18 (29.0%)	0.280
Metabolic	29 (15.3%)	18 (14.1%)	11 (17.7%)	0.509
Infection	9 (4.7%)	7 (5.5%)	2 (3.2%)	0.495
Reason for coming off				0.049
Died or Poor Prognosis	48 (21.1%)	26 (16.4%)	22 (32.4%)	
ECLS Complication	16 (7.0%)	11 (6.9%)	5 (7.4%)	
Expected Recovery	153 (67.4%)	116 (73.0%)	37 (54.4%)	
Unknown	1 (0.4%)	1 (0.6%)	0 (0.0%)	
VAD	9 (4.0%)	5 (3.1%)	4 (5.9%)	
Missing	2	2	0	
AKI Stage II or III	160 (75.8%)	111 (74.5%)	49 (79.0%)	0.483
Mortality	102 (44.5%)	65 (40.4%)	37 (54.4%)	0.050

Results Depicted in *n* (%), and Median (Interquartile Range/IQR). ECLS: Extracorporeal Life Support; VA: Veno-Arterial; VAD: Ventricular Assist Device; AKI: Acute Kidney Injury.

### Cut-point analysis

**Figure 1:**
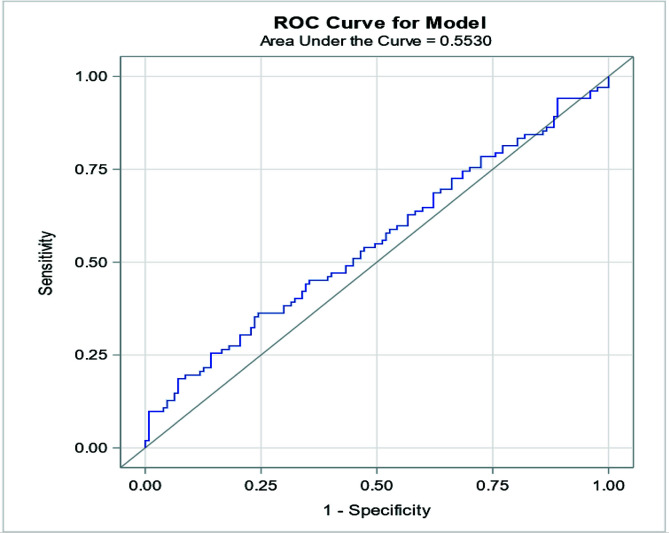
Receiver operating characteristic (ROC) curve identifying the optimal discriminatory cut point for mortality was 233 mmHg (sensitivity 36%, specificity 76%).

**Figure 2:**
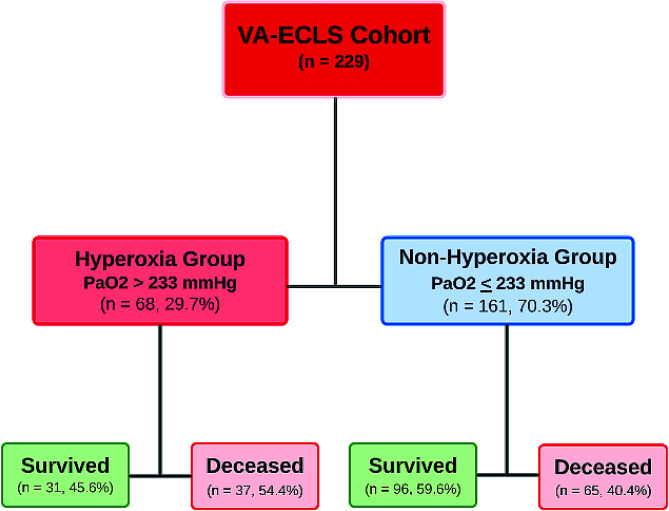
Flow chart of neonates requiring Veno-arterial Extracorporeal Life Support (VA-ECLS) stratified based on PaO_2_ levels in the first 48 h while on ECLS.

Using ROC analysis, PaO_2_ > 233 mmHg had the optimal discriminatory ability for mortality with a sensitivity of 36%, and specificity of 76%, and was defined as hyperoxia for this study population (Fig. 1). AUC for PaO_2_ to predict mortality was 0.55 (95% CI [0.47–0.62]; *p* = 0.212). Approximately one-third of the patients were in the hyperoxia group (68/229) with the remainder in the non-hyperoxia group (161/229) (Fig. 2). The two cohorts were similar in demographic variables [i.e., weight, height, body surface area (BSA), sex, and race/ethnicity] except for age (Table 1). Patients in the hyperoxia group were older [4.6 months (IQR 0.4, 27.8) vs 1.5 months (IQR 0.2, 17.2), *p* = 0.019] and had higher median initial ECLS flow in the first 2 h [128 mL/kg/min (IQR 97, 131) vs 109 mL/kg/min (IQR 100, 121), *p* = 0.018]. The most common indication to require ECLS (based on ELSO registry criteria) was cardiac in both groups (60.3% and 44.1%, respectively). Patients in the hyperoxia group had a lower rate of expected recovery and ability to wean off ECLS (54.4% vs 73%, *p* = 0.049). Compared to the non-hyperoxia group, we found that mortality was higher in the hyperoxia group (54.4% vs 40.45, *p* = 0.050) (Table 1, Fig. 2).

### Outcomes analysis

**Figure 3:**
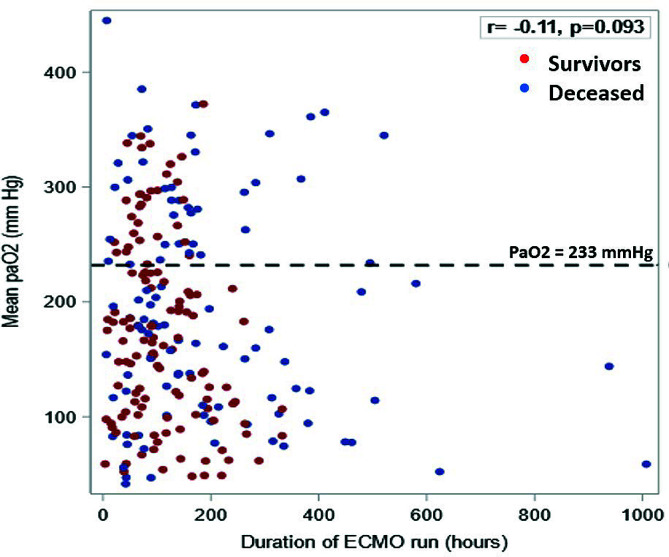
Scatterplot illustrating the relationship of average PaO_2_, VA-ECLS run duration, and mortality in the VA-ECLS Cohort.

In the univariable analysis, we found that the hyperoxia group had increased odds of mortality (OR 1.76, 95% CI [0.995–3.12], *p* = 0.052). In the multivariable analysis when controlling for age group (neonates vs pediatrics) and indication for ECLS, hyperoxia continued to demonstrate higher odds of mortality (aOR 2.02, 95% CI [1.03–3.97], *p* = 0.03) and higher odds of developing stage II or III AKI (aOR 2.12 95% CI [0.82–5.50], *p* = 0.12) (Table 2), but this did not reach statistical significance. The association of average PaO_2_ and ECLS duration is graphically demonstrated in Figure 3 (correlation coefficient −0.11, 95% CI [−0.33 – 0.03], *p* = 0.093).

**Table 2:** Outcomes of patients undergoing VA-ECLS using a univariable and multivariable regression analysis.

Variables	Non-Hyperoxia Group	Hyperoxia Group	OR (95% CI)	*p*-value	aOR^a^ (95% CI)	Adjusted *p*-value
	PaO_2_ ≤ 232 mmHg (n = 161)	PaO_2_ > 232 mmHg (n = 68)				
Mortality	65 (40.4%)	37 (54.4%)	1.76 (0.995–3.12)	0.052	2.02 (1.03–3.97)	0.03
Any Cardiovascular, Renal, or Mechanical complication	113 (70.2%)	54 (79.4%)	1.29 (0.63–2.64)	0.478	1.96 (0.89–4.35)	0.10
Stage II/III AKI**	111 (74.5%)	49 (79.0%)	1.64 (0.83–3.23)	0.154	2.12 (0.82–5.50)	0.12

a Adjusted for age (months) and mode of VA-ECLS.

### Functional Status Scale (FSS) of survivors and development of new morbidity and unfavorable outcomes

Among the non-hyperoxia group, 21 of the 96 survivors (21.9%) developed new morbidity along with 9 out of 31 survivors (29.0%) of the hyperoxia group. Unfavorable functional outcome (defined as an increase in FSS ≥ 5) occurred in 8/96 patients in the non-hyperoxia survivors (8.3%), and 2/31 (6.5%) in the non-hyperoxia group. We failed to identify an association between designation as “hyperoxia” and new morbidity or unfavorable outcomes (Table 3).

**Table 3:** New morbidity and unfavorable functional outcome for overall survivors who required VA-ECLS stratified by PaO_2_ levels into hyperoxia and non-hyperoxia groups based on functional status scale change from admission to discharge.

ECLS Group	Overall cohort of survivors (*n* = 127)	Non-hyperoxia group	Hyperoxia group	*p*-value
		PaO_2_ ≤ 233 mmHg (*n* = 96)	PaO_2_ > 233 mmHg (*n* = 31)	
New morbidity (Change in FSS score ≥3 points)	30 (23.6%)	21 (21.9%)	9 (29.0%)	0.415
Unfavorable outcome (Change in FSS score ≥5 points)	10 (7.9%)	8 (8.3%)	2 (6.5%)	0.735

FSS Subscale scores range from 1 to 5. Total scores are the sum of subscale scores, ranging from 6 to 30.

FSS: Functional Status Scale; ECLS: Extracorporeal Life Support.

### Subset analysis of patients with a cardiac diagnosis who required ECLS post-cardiotomy

There were a total of 196 patients with a cardiac diagnosis who required VA-ECLS. Of those, 155 were post-cardiotomy and are shown in Supplemental Table 1. We stratified the post-cardiotomy patients using the PaO_2_ cut point that was used above (PaO_2_ 233 mmHg) and found that patients in the hyperoxia group had a longer duration of ECLS run (149 vs 88 h, *p* = 0.0004), and had a lower rates of expected recovery (66.1 vs 78.1, *p* = 0.027). The full comparison between the cohorts is shown in Supplemental Table 2.

## Discussion

In this single-center retrospective report from a high-volume ECLS center, we describe an overall mortality rate of patients supported on VA-ECLS of 44.5%. Using a ROC curve, a mean PaO_2_ of 233 mmHg in the first 48 h of ECLS was determined to have the optimal discriminatory ability for mortality (sensitivity 36% and specificity 76%). Of the VA-ECLS runs, 68 (29.7%) had mean PaO_2_ > 233 mmHg and were categorized as the hyperoxia group. Patients in the hyperoxia group tended to be older (median age 4.6 vs 1.5 months, *p* = 0.019), to have a cardiac indication for VA-ECLS (60% vs 44%, *p* = 0.0004), and had lower rates of expected recovery to come off ECLS (54% vs 73%, *p* = 0.049). Multi-variable logistic regression controlling for confounding variables demonstrated a suggestive association, though it failed to reach statistical significance, between hyperoxia and mortality in the unadjusted analysis (OR 1.76, 95% CI [0.995–3.12], *p* = 0.052). While hyperoxia during ECLS did not directly lead to death, we postulate that it may contribute to new morbidities, such as acute kidney injury, that would later lead to complications and mortality.

In other critical illness settings, an association between excessive oxygen delivery and poor clinical outcomes has been reported. In patients requiring ECLS post-cardiac arrest, hyperoxia (as defined by a mean PaO_2_ > 193 mmHg) was associated with 30-day mortality and the need for dialysis [4, 15, 25]. In a large multicenter cohort study of adult patients admitted to the ICU after resuscitation from cardiac arrest (CA), Kilgannon *et al.* showed an association between hyperoxia and risk of in-hospital death consistent with a dose-dependent relationship [15]. In a prospective disease-specific CA database, Elmer and colleagues found that exposure to severe hyperoxia was independently associated with inpatient mortality [25]. A retrospective study by Al-Kawaz and colleagues demonstrated poor neurological outcomes in patients exposed to mild, moderate, and severe hyperoxia in the first 24 h of ECLS. Additional duration of severe hyperoxia was independently associated with in-hospital mortality [13]. Several reports of neonates with asphyxia have demonstrated an association between hyperoxia and an increased risk of brain injury and mortality [4, 26, 27]. Conversely, Raman *et al*, in a single-center study and systematic review of a heterogeneous cohort of critically ill patients, did not demonstrate an association between hyperoxia at the time of admission and mortality [28].

In a prior report, we showed that a substantial portion of infants undergoing cardiac surgery using CPB were exposed to hyperoxia and that patients in the hyperoxia group had four-fold greater odds of mortality within 30 days of surgery [9]. This report supports earlier findings that hyperoxia is likely associated with worse outcomes, but which populations are at risk remains unclear, and the impact of other clinical variables that may affect oxygenation in a direct or indirect way. Some of these factors are patient hemoglobin levels, ventilator settings including FiO_2_, the health and age of the oxygenator in the ECLS circuit, ECLS flows, recirculation, and whether the patient is sedated and paralyzed to decrease oxygen consumption. It would be extremely useful to control these factors, but in reality, the degree of impact of each factor is different for each patient. This really supports the importance of this study and future studies to help understand the true impact of oxygen on patient outcomes and on the biological systems of the body. However, the delineation of risk categories in different patient populations remains unclear.

There is no generally accepted value to discriminate pathologic hyperoxia. Injurious hyperoxia may vary by patient population and clinical context [25]. Poor outcomes may occur when PaO_2_ exceeds a certain threshold, where endogenous antioxidants are unable to prevent oxidative stress. When high amounts of oxygen are introduced to previously ischemic tissues, this leads to the generation of reactive oxygen species (ROS) and activation of inflammatory pathways via cytokines and other immunological signaling pathways [29]. The generation of ROS leads to lipid peroxidation and protein changes, resulting in cell injury. In patients who have experienced CA or resuscitation aftershock, an increase in ROS may deplete plasma antioxidant potential, which may lower the threshold for subsequent oxidative injury [14, 25], and this effect may be more pronounced in neonates and infants due to immature antioxidant defenses [4]. The effect of relative hyperoxia may be even more pronounced in patients with cyanotic heart disease who have significantly lower baseline PaO_2_. It is not known if the antioxidant systems of the body are downregulated in patients with chronically lower baseline PaO_2_. When placed on ECLS, these patients are exposed to a relative hyperoxia state. It is plausible that these patients are more vulnerable to supraphysiologic oxygen, the extent of which is yet unknown.

Because there is no generally accepted definition of hyperoxia in neonates with congenital heart disease with various physiology and complexity in different settings, we used a ROC curve analysis in this specific cohort to determine which PaO_2_ values may be associated with adverse outcomes. This similar strategy was employed by Sznycer-Taub *et al.*, and Beshish *et al.* in two separate reports. Sznycer-Taub and colleagues evaluated hyperoxia in pediatric cardiac patients (neonates and infants) supported on VA-ECLS and found that a PaO_2_ of 193 mmHg in the first 48 h was determined to have good discriminatory ability about 30-day mortality [4]. Using a similar strategy, Beshish and colleagues showed that a PaO_2_ of 313 mmHg for infants undergoing cardiac surgery utilizing CPB was independently associated with 30-day mortality [9]. Our cut-off definition of hyperoxia was very close to that identified by Sznycer-Taub *et al.* although the patient population that was used by Sznycer-Taub was slightly different, as the former study captured all infants who required VA-ECLS following cardiac surgery. In that study, the cutoff point remained the same (PaO_2_ of 193 mmHg) in a subgroup analysis of all neonates (*n* = 70), and in neonates who underwent a Norwood operation (*n* = 35).

## Limitations

Our findings are subject to all limitations inherent to single-center retrospective cohort studies. Although samples to measure PaO_2_ were obtained at dedicated time intervals, it is not possible to discern the effect of time spent in a hyperoxia state as opposed to the effects of acutely high PaO_2_ levels. Additionally, there may be some bias as to which patients are exposed to hyperoxia. The majority of our cohort had a PaO_2_ level around or over 200 mmHg while on VA-ECLS, which limited our ability to study the relationship between lower oxygen tension levels and outcomes. Although we identified a cut point for PaO_2_ of 233 mmHg using an AUC, the sensitivity was 36%. The sensitivity is low, and this is clearly a limitation of our study that we think can be overcome with a larger patient population that is more homogenous. Importantly, many of these limitations can be addressed in a multicenter validation study, which our group is currently pursuing. It is acceptable that the AUC for the model is low, which to the complexity of hyperoxia, which might not be properly captured by the variables in the model. There is also the possibility of interaction between predictors, which was not accounted for. An alternative way that can be done in future analyses is to combine different sets of predictors or explore other analytic approaches that might be able to yield strong associations within certain patient populations. Also, when presenting findings in Figure 3, we show that the correlation between PaO_2_ level, duration of ECLS and mortality was not significant. This could be related to many factors such as the heterogeneous patient population, patient response to ECLS exposure, severity of illness, and other Confounding factors, which can also play a crucial role, such that the important predictors might not have been accounted for in the correlation, to name a few.

## Conclusions

Of the 229 VA-ECLS runs in 209 patients, hyperoxia during the first 48 h of VA-ECLS defined by a mean PaO_2_ > 233 mmHg, occurred in approximately 30% of runs. Patients in the hyperoxia group were older and had higher median initial ECLS flow in the first 2 h. The mortality rate was higher in the hyperoxia group, and although it did not reach statistical significance, patients in the hyperoxia group had higher odds of mortality (*p* = 0.052). Multicenter and prospective evaluation of this modifiable risk factor is imperative to improve the care of this high-risk cohort.

## Data Availability

All available data are incorporated into the article.

## Supplementary material

The supplementary material is available at https://ject.edpsciences.org/10.1051/ject/2025006/olm. *Supplemental Table 1*. Cardiac Diagnosis of Patient in the VA-ECLS Cohort. *Supplemental Table 2*. Patient Demographics and Clinical Characteristics of Patients Who Required VA-ECLS Post-Cardiotomy Stratified by Mean PaO_2_ Levels in the First 48 h into Non-Hyperoxia Group (PaO_2_ ≤ 233 mmHg), Hyperoxia Group (PaO_2_ > 233 mmHg).

## References

[1] Munshi L, Kiss A, Cypel M, Keshavjee S, Ferguson ND, Fan E. Oxygen thresholds and mortality during extracorporeal life support in adult patients. Crit Care Med. 2017;45(12):1997–2005. 28787294 10.1097/CCM.0000000000002643

[2] Raffaeli G, Ghirardello S, Passera S, Mosca F, Cavallaro G. Oxidative stress and neonatal respiratory extracorporeal membrane oxygenation. Front Physiol. 2018;9:1739. 30564143 10.3389/fphys.2018.01739 PMC6288438

[3] ELSO. Registry of the extracorporeal life support organization. Ann Arbor ME: Registry Report; 2024.

[4] Sznycer-Taub NR, Lowery R, Yu S, Owens ST, Hirsch-Romano JC, Owens GE. Hyperoxia is associated with poor outcomes in pediatric cardiac patients supported on venoarterial extracorporeal membrane oxygenation. Pediatr Crit Care Med. 2016;17(4):350–358. 27043897 10.1097/PCC.0000000000000655

[5] Cashen K, Reeder R, Dalton HJ, et al. Hyperoxia and hypocapnia during pediatric extracorporeal membrane oxygenation: associations with complications, mortality, and functional status among survivors. Pediatr Crit Care Med. 2018;19(3):245–253. 29319634 10.1097/PCC.0000000000001439 PMC5834382

[6] Ni YN, Wang YM, Liang BM, Liang ZA. The effect of hyperoxia on mortality in critically ill patients: a systematic review and meta analysis. BMC Pulm Med. 2019;19(1):53. 30808337 10.1186/s12890-019-0810-1 PMC6390560

[7] Bonnemain J, Rusca M, Ltaief Z, et al. Hyperoxia during extracorporeal cardiopulmonary resuscitation for refractory cardiac arrest is associated with severe circulatory failure and increased mortality. BMC Cardiovasc Disord. 2021;21(1):542. 34775951 10.1186/s12872-021-02361-3 PMC8591834

[8] Brown DM, Holt DW, Edwards JT, Burnett RJ. 3rd: Normoxia vs. hyperoxia: impact of oxygen tension strategies on outcomes for patients receiving cardiopulmonary bypass for routine cardiac surgical repair. J Extra Corpor Technol. 2006;38(3):241–248. 17089511 PMC4680816

[9] Beshish AG, Jahadi O, Mello A, Yarlagadda VV, Shin AY, Kwiatkowski DM. Hyperoxia during cardiopulmonary bypass is associated with mortality in infants undergoing cardiac surgery. Pediatr Crit Care Med. 2021;22(5):445–453. 33443979 10.1097/PCC.0000000000002661

[10] Lilien TA, Groeneveld NS, van Etten-Jamaludin F, et al. Association of arterial hyperoxia with outcomes in critically Ill children: a systematic review and meta-analysis. JAMA Netw Open. 2022; 5(1):e2142105. 34985516 10.1001/jamanetworkopen.2021.42105 PMC8733830

[11] Stoll SE, Paul E, Pilcher D, Udy A, Burrell A. Hyperoxia and mortality in conventional versus extracorporeal cardiopulmonary resuscitation. J Crit Care. 2022;69:154001. 35217372 10.1016/j.jcrc.2022.154001

[12] McDonald CI, Fraser JF, Coombes JS, Fung YL. Oxidative stress during extracorporeal circulation. Eur J Cardiothorac Surg. 2014;46(6):937–943. 24482384 10.1093/ejcts/ezt637

[13] Al-Kawaz MN, Canner J, Caturegli G, et al. Duration of hyperoxia and neurologic outcomes in patients undergoing extracorporeal membrane oxygenation. Crit Care Med. 2021;49(10):e968–e977. 33935164 10.1097/CCM.0000000000005069

[14] Chang WT, Wang CH, Lai CH, et al. Optimal arterial blood oxygen tension in the early postresuscitation phase of extracorporeal cardiopulmonary resuscitation: A 15-year retrospective observational study. Crit Care Med. 2019;47(11):1549–1556. 31356478 10.1097/CCM.0000000000003938

[15] Kilgannon JH, Jones AE, Parrillo JE, et al. Relationship between supranormal oxygen tension and outcome after resuscitation from cardiac arrest. Circulation. 2011;123(23):2717–2722. 21606393 10.1161/CIRCULATIONAHA.110.001016

[16] Page D, Ablordeppey E, Wessman BT, et al. Emergency department hyperoxia is associated with increased mortality in mechanically ventilated patients: a cohort study. Crit Care. 2018;22(1):9. 29347982 10.1186/s13054-017-1926-4 PMC5774130

[17] Davis DP, Meade W, Sise MJ, et al. Both hypoxemia and extreme hyperoxemia may be detrimental in patients with severe traumatic brain injury. J Neurotrauma. 2009;26(12):2217–2223. 19811093 10.1089/neu.2009.0940

[18] Stevens PE, Levin A. Evaluation and management of chronic kidney disease: synopsis of the kidney disease: improving global outcomes 2012 clinical practice guideline. Ann Intern Med. 2013;158(11):825–830. 23732715 10.7326/0003-4819-158-11-201306040-00007

[19] Pollack MM, Holubkov R, Glass P, et al. Functional Status Scale: new pediatric outcome measure. Pediatrics. 2009;124(1):e18–e28. 19564265 10.1542/peds.2008-1987 PMC3191069

[20] Berg RA, Nadkarni VM, Clark AE, et al. Incidence and outcomes of cardiopulmonary resuscitation in PICUs. Crit Care Med. 2016;44(4):798–808. 26646466 10.1097/CCM.0000000000001484 PMC4809365

[21] Beshish AG, Baginski MR, Johnson TJ, Deatrick BK, Barbaro RP, Owens GE. Functional status change among children with extracorporeal membrane oxygenation to support cardiopulmonary resuscitation in a pediatric cardiac ICU: a single institution report. Pediatr Crit Care Med. 2018;19(7):665–671. 29659415 10.1097/PCC.0000000000001555

[22] Beshish AG, Rodriguez Z, Hani Farhat M, et al. Functional status change among infants, children, and adolescents following extracorporeal life support: a multicenter report. ASAIO J. 2022;69(1):114–121. 35435861 10.1097/MAT.0000000000001711

[23] Han B, Yang JK, Ling AY, et al. Early functional status after surgery for congenital heart disease: a single-center retrospective study. Pediatr Crit Care Med. 2021;23(2):109–117. 10.1097/PCC.0000000000002838 34593740

[24] Pollack MM, Holubkov R, Funai T, et al. Relationship between the functional status scale and the pediatric overall performance category and pediatric cerebral performance category scales. JAMA Pediatr. 2014;168(7):671–676. 24862461 10.1001/jamapediatrics.2013.5316 PMC4589215

[25] Elmer J, Scutella M, Pullalarevu R, et al. The association between hyperoxia and patient outcomes after cardiac arrest: analysis of a high-resolution database. Intensive Care Med. 2015;41(1):49–57. 25472570 10.1007/s00134-014-3555-6 PMC4337386

[26] Klinger G, Beyene J, Shah P, Perlman M. Do hyperoxaemia and hypocapnia add to the risk of brain injury after intrapartum asphyxia? Arch Dis Child Fetal Neonatal Ed. 2005;90(1):49–52. 10.1136/adc.2003.048785 PMC1721814 15613575

[27] Rabi Y, Rabi D, Yee W. Room air resuscitation of the depressed newborn: a systematic review and meta-analysis. Resuscitation. 2007;72(3):353–363. 17240032 10.1016/j.resuscitation.2006.06.134

[28] Raman S, Prince NJ, Hoskote A, Peters MJ. Admission PaO_2_ and mortality in critically ill children: a cohort study and systematic review. Pediatr Crit Care Med. 2016;17(10):e444–e450. 27509363 10.1097/PCC.0000000000000905

[29] Turer AT, Hill JA. Pathogenesis of myocardial ischemia-reperfusion injury and rationale for therapy. Am J Cardiol. 2010;106(3):360–368. 20643246 10.1016/j.amjcard.2010.03.032 PMC2957093

