# Supplementary material for: Is hyperoxia during veno-arterial extracorporeal life support due to cardiopulmonary failure associated with mortality in pediatric patients?
Source: J Extra Corpor Technol. 2025 Sep 15;57(3):129–36. doi: 10.1051/ject/2025006 (PMC12435806; doi:10.1051/ject/2025006)
Supplement: Supplementary file 1 — Supplemental Table 1. Cardiac Diagnosis of Patient in the VA-ECLS Cohort. Supplemental Table 2. Patient Demographics and Clinical Characteristics of Patients Who Required VA-ECLS Post-Cardiotomy Stratified by Mean PaO2 Levels in the First 48 h into Non-Hyperoxia Group (PaO2 ≤ 233 mmHg), Hyperoxia Group (PaO2 > 233 mmHg). [file ject-57-129-s1.pdf]

Supplemental – Table 1. Cardiac Diagnosis of Patient in the VA-ECLS Cohort.

**Cardiac Diagnosis Description**	**(n = 196)**
**POSTCARDIOTOMY (n = 155)**	
Single Ventricle Physiology	34
Pulmonary Stenosis (Valvar, Supra Valvar and branch pulmonary arteries stenosis)	24
Coarctation of the Aorta and Aortic Arch Abnormalities	20
Atrioventricular Septal Defect	10
Transposition of the Great Arteries (L-TGA & D-TGA +/- VSD)	10
Double Outlet Right Ventricle	10
Atrial Septal Defect	10
Ventricular Septal Defect	9
Pulmonary Vein Abnormalities	7
Heterotaxy Syndrome	7
Tetralogy of Fallot	6
Coronary Abnormalities	6
Cardiac Conduction Abnormality	2
**NON-POSTCARDIOTOMY (n = 41)**	
Cardiomyopathy	3
Other Diagnosis	38

Supplemental – Table 2. Patient Demographics and Clinical Characteristics of Patients who Required VA-ECLS Post-Cardiotomy Stratified by Mean PaO_2 Levels in the First 48-hours into Non-Hyperoxia Group ($PaO_2 \leq 233$ mmHg) Hyperoxia Group ($PaO_2 > 233$ mmHg)

**Variables**	**Total Cohort (n = 155)**	**Non-Hyperoxia Group ($PaO_2 \leq$ 233 mmHg) (n = 96)**	**Hyperoxia Group (PaO_2 233 mmHg) (n = 59)**	**p-value**
**Age (months)**	1.5 (0.4, 6.3)	1.0 (0.4, 5.3.	1.5 (0.4, 6.8)	0.555
**Age Group**	137 (88.4%)	85 (88.5%)	52 (88.1%)	0.939
Neonatal	18 (11.6%)	11 (11.5%)	7 (11.9%)	
Pediatrics				
**Weight (kg)**	3.9 (2.9, 6.1)	4.0 (3.1, 6.1)	3.4 (2.7, 6.0)	0.3924
**Height (cm)**	52.0 (48.0, 62.0)	52.0 (48.0, 60.0)	51.0 (49.0, 64.0)	0.6501
**BSA (m^2)**	0.2 (0.2, 0.3)	0.2 (0.2, 0.3)	0.2 (0.2, 0.3)	0.9921
**Race**	70 (45.2%)	47 (49.0%)	23 (39.0%)	0.452
Black	23 (14.8%)	15 (15.6%)	8 (13.6%)	
White	56 (36.1%)	30 (31.3%)	26 (44.1%)	
Hispanic	6 (3.9%)	4 (4.2%)	2 (3.4%)	
Other				
**Sex**	71 (45.8%)	43 (44.8%)	28 (47.45)	0.746
Female	84 (54.2%)	53 (55.2%)	31 (52.5%)	
Male				
**ECLS Indication**	108 (69.7%)	66 (68.8%)	42 (71.2%)	0.749
Cardiac	47 (30.3%)	30 (31.3%)	17 (28.8%)	
ECPR				
**Time from admission to ECLS Initiation**	114.0 (28.0, 363.0)	76.0 (26.0, 406.0)	152.0 (41.0, 359.0)	0.101
**Initial ECLS flow (ml/kg/min)**	0.5 (0.4, 0.7)	0.5 (0.3, 0.7)	0.5 (0.4, 0.7)	0.4281
**Duration of ECLS run (hours)**	89.0 (50.0, 161.0)	88.0 (42.0, 139.0)	149.0 (72.0, 172.0)	**0.0004**

**ECLS Complications:**	89 (65.4%)	55 (69.6%)	34 (59.6%)	0.228
**Hemorrhagic**	28 (20.6%)	16 (20.3%)	12 (21.1%)	0.909
**mechanical**	77 (56.6%)	42 (53.2%)	35 (61.4%)	0.339
**Renal**	31 (22.8%)	15 (19.0%)	16 (28.1%)	0.213
**Neurologic**	18 (13.2%)	11 (13.9%)	7 (12.3%)	0.780
**metabolic**	4 (2.9%)	3 (3.8%)	1 (1.8%)	0.487
**Infection**				
**Reason for coming off**	32 (20.6%)	49 (19.8%)	13 (22.0%)	**0.027**
Died or Poor Prognosis	5 (3.2%)	0 (0.0%)	5 (8.5%)	
ECLS Complication	114 (73.5%)	75 (78.1%)	39 (66.1%)	
Expected Recovery	4 (2.6%)	2 (2.1%)	2 (3.4%)	
VAD				
**AKI Stage II or III**	121 (84.6%)	73 (83.0%)	48 (87.3%)	0.486
**Mortality**	81 (52.3%)	48 (50.0%)	33 (55.9%)	0.473

Results Depicted in n (%), and Median (Interquartile Range/IQR)

ECLS: Extracorporeal Life Support; VA: Veno-Arterial; VAD: Ventricular Assist Device; AKI: Acute Kidney Injury.
